# Crosstalk between Calcium and ROS Signaling during Flg22-Triggered Immune Response in *Arabidopsis* Leaves

**DOI:** 10.3390/plants11010014

**Published:** 2021-12-21

**Authors:** Matthew J. Marcec, Kiwamu Tanaka

**Affiliations:** 1 Department of Plant Pathology, Washington State University, Pullman, WA 99164, USA; matthew.marcec@wsu.edu; 2 Molecular Plant Sciences Program, Washington State University, Pullman, WA 99164, USA

**Keywords:** pattern-triggered immunity (PTI), second messengers, cytosolic calcium, reactive oxygen species (ROS), respiratory burst oxidase homologs (RBOHs)

## Abstract

Calcium and reactive oxygen species (ROS) are two of the earliest second messengers in response to environmental stresses in plants. The rise and sequestration of these messengers in the cytosol and apoplast are formed by various channels, transporters, and enzymes that are required for proper defense responses. It remains unclear how calcium and ROS signals regulate each other during pattern-triggered immunity (PTI). In the present study, we examined the effects of perturbing one signal on the other in *Arabidopsis* leaves upon the addition of flg22, a well-studied microbe-associated molecular pattern (MAMP). To this end, a variety of pharmacological agents were used to suppress either calcium or ROS signaling. Our data suggest that cytosolic calcium elevation is required to initiate and regulate apoplastic ROS production generated by respiratory burst oxidase homologs (RBOHs). In contrast, ROS has no effect on the initiation of the calcium signal, but is required for forming a sufficient amplitude of the calcium signal. This finding using pharmacological agents is corroborated by the result of using a genetic double mutant, *rbohd rbohf*. Our study provides an insight into the mutual interplay of calcium and ROS signals during the MAMP-induced PTI response in plants.

## 1. Introduction

Plants, like all organisms, depend on the ability to perceive and respond to environmental challenges. Discriminating between the myriad of different stresses that plants are exposed to is dependent on signaling systems based around second messenger molecules. Second messengers include molecules such as reactive oxygen species (ROS), cytosolic free calcium, nitric oxide, cyclic nucleotides, and phospholipids. Upon an environmental stimulus, the second messengers rise in concentration in the cytosol and apoplast and are quickly sequestered due to their often toxic nature, making them excellent signaling molecules [1,2,3]. It has been observed that different stimuli often produce a unique rise and fall in the concentration of secondary massagers, thus dubbing them “signatures” [3]. Each signature dynamics must be unperturbed for the plant to invoke the correct response to environmental stresses [4].

In plant innate immune responses, these signatures are triggered as early cellular responses when pattern recognition receptors (PRRs) are activated by molecular patterns derived from pathogens and the damaged plants themselves, so-called MAMPs and DAMPs, respectively [5,6]. For example, cells damaged by wounding and herbivory release ATP to extracellular spaces, e.g., the apoplast [7,8,9]. Adjacent cells sense the released ATP as a DAMP via the PRR P2K1/DORN1 [10], evoking a specific calcium signature [11,12]. This calcium signature is accompanied by ROS, NO and electric signatures that all orchestrate specific plant responses, such as pattern-triggered immunity (PTI) [13,14,15,16,17]. Likewise, MAMPs such as flagellin 22 (flg22), which is sensed by the PRR FLS2, lead to canonical PTI responses [18,19]. Evidence suggests that activation of PRRs induces specific signatures of various second messengers that contribute to an appropriate qualitative and quantitative immune response.

Two of the most well-studied second messengers in plant immunity are calcium and ROS. Cytosolic calcium signal in plants is formed by various types of calcium channels in the plasma membrane and in organelles, such as the vacuole and chloroplast [20]. Calcium channels found in plants include hyperpolarization-activated calcium channels (HACCs) and depolarization-activated calcium channels (DACCs), although only limited information regarding genetic evidence of these channel types is available in plants [21]. Mathematical models have been developed to describe how calcium signatures are generated based on action potentials or through calcium ion fluxes [22,23]. Potential HACCs have been found in the vacuole (TPC1), the plasma membrane via cyclic nucleotide channels (CNGCs), glutamate receptor-like genes (GLRs) and osmosensitive calcium permeable cation channels (OSCAs) [24,25,26,27]. GLR receptors have been shown to regulate leaf-to-leaf calcium signature propagation [26,28,29]. The slow vacuolar cation channel TPC1 [24] fulfills mathematical calcium signature models and offers a feasible explanation that calcium signaling requires a slow acting channel [28]. Cyclic nucleotide gated channels (CNGCs) are also involved in forming calcium signatures and have been implicated in abiotic and biotic stress responses [25,30]. The OSCA1.3 channel in *Arabidopsis* specifically interacts with stomatal guard cells in plant immune responses [27].

During stress responses in plants, ROS are propagated from respiratory burst oxidase homologs (RBOHs) that act as NADPH oxidases (NOXs). In Arabidopsis, RbohD and RbohF [31,32,33] mainly generate ROS upon activation of PRRs: plants lacking both RBOH proteins fail to generate ROS in response to biotic stresses [34]. The ROS signature produced by RbohD and RbohF is regulated by PRR complexes upon recognition of MAMPs and DAMPs of all kinds [34]. Recent studies revealed that the FLS2-associated kinase BIK1 directly phosphorylates N-terminal serine residues of RbohD, thereby, RbohD activation followed by ROS propagation is induced in PTI [35,36].

How ROS and calcium signals interact with and regulate each other is still unclear. Studies show that RbohD and RbohF are dependent on calcium signals due to N terminal EF hands, which are calcium-binding motifs [32,37]. In addition, there are phosphorylation sites on the N terminal domain, which are targeted by calcium-dependent protein kinases (CPKs) for activation. The serine residues at S39, S148, S163 and S347 on RbohD are phosphorylated in the CPK5 overexpression lines [38]. Notably, the CPK5 overexpression lines showed consistently enhanced ROS production via RbohD, while its knockout mutant had limited ROS production in comparison to the wild type [39]. These data suggest that calcium signaling is necessary for the activation of RBOHs to induce ROS production. In contrast, ROS are known to affect the propagation of calcium signaling. For example, ROS-induced cytosolic calcium elevation is observed during many plant responses, e.g., stomatal closure and programmed cell death [40,41,42]. Although there is little information regarding the underlying mechanism, ROS-induced cytosolic calcium elevation is likely due to the direct regulation of calcium channels [43,44]. One can speculate that these calcium channels can be regulated by ROS in a redox-dependent manner. Although available information demonstrated by the studies described above has contributed to elucidating the mechanisms, it remains unclear how calcium and ROS signals regulate each other during PTI response.

To explore how calcium and ROS signaling interact during the PTI response, the present study evaluated the effect of perturbation of one signature on the other in *Arabidopsis*. To this end, we pharmacologically perturbed calcium and ROS signatures using well-known inhibitors and scavengers of both second messengers in a dose-dependent manner to gain a clear picture of how calcium and ROS affect one another in plant immunity, where the flg22-induced calcium/ROS response was used as a model system. We also used a genetic material, *rbohd/f* double mutant to evaluate the contribution of flg22-induced ROS signal to cytosolic calcium signaling. Cytosolic calcium was measured using an aequorin-based bioluminescent assay, while apoplastic ROS was measured using a luminol-based chemiluminescence assay, as both have been shown to be robust methods for measuring these signatures [42,45]. We found that ROS was required for tuning, but not for initiation of calcium signaling, while calcium was required for both initiation and tuning of ROS signaling. Our data provide a clearer picture of how ROS and calcium signatures are required for proper dynamics for one another.

## 2. Results

**Figure 1:**
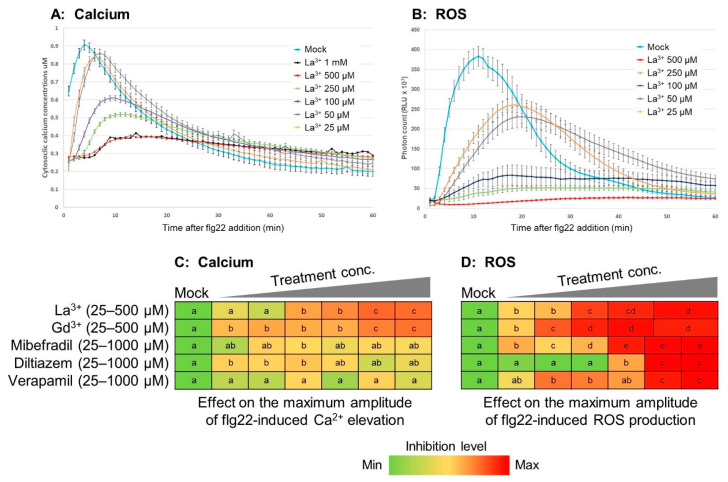
Effects of calcium manipulation on apoplastic ROS signaling. Line graphs show effect of the calcium channel blocker La^3+^ on flg22-induced cytosolic calcium signature (**A**) and flg22-induced apoplastic ROS signature (**B**). Heatmaps show the inhibitory effect of pharmacological agents on the maximum amplitude of flg22-induced cytosolic calcium elevation (**C**) and flg22-induced apoplastic ROS production (**D**). Leaf discs were preincubated for 30 min with general calcium channel blockers La^3+^ and Gd^3+^. (25–500 µM), the VDCC specific inhibitors mibefradil, diltiazem, and Verapamil (25–1000 µM) prior to flg22 addition. Colors indicate the lowest level of inhibition as green and the highest level of inhibition as red. Different letters in the same row indicate statistically significant differences at *p* < 0.05. The data obtained from the maximum amplitude in each treatment were analyzed using ANOVA followed by the Tukey–Kramer multiple comparison test.

**Figure 2:**
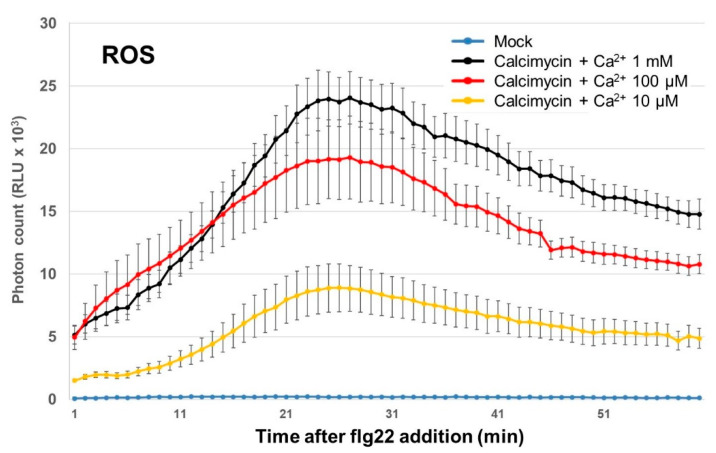
Direct effect of increased cytosolic calcium on apoplastic ROS signaling under exposure to a calcium ionophore. Line graph shows the effects of adding calcium (as 10 µM, 100 µM, or 1 mM of CaCl_2_) after incubation with the calcium ionophore calcimycin (100 µM) on apoplastic ROS production. Note that ROS productions are raised by addition of calcium in a dose-dependent manner.

**Figure 3:**
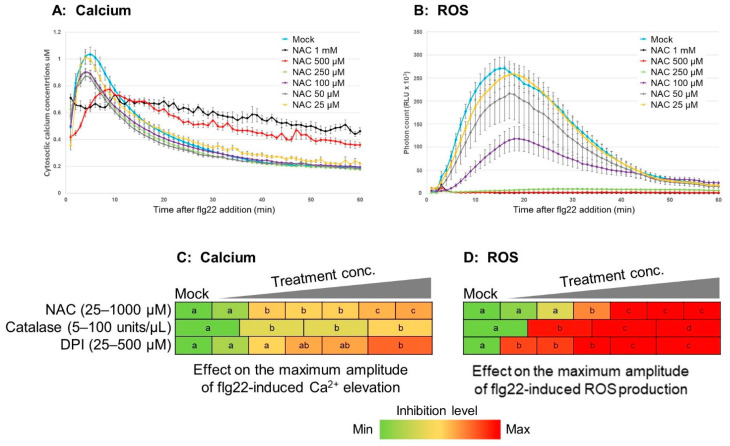
Effects of ROS manipulation on cytosolic calcium signaling. Line graphs show effect of the ROS scavenger NAC on flg22-induced cytosolic calcium signature (**A**) and flg22-induced apoplastic ROS signature (**B**). Heatmaps show the inhibitory effect of pharmacological agents on the maximum amplitude of flg22-induced cytosolic calcium elevation (**C**) and flg22-induced apoplastic ROS production (**D**). Leaf discs were preincubated for 30 min with the ROS scavengers NAC (25–1000 µM) and catalase (5–100 units/uL) and the NADPH oxidase inhibitor DPI (25–500 µM) prior to flg22 addition. Colors indicate the lowest level of inhibition as green and the highest level of inhibition as red. Different letters in the same row indicate statistically significant differences at *p* < 0.05. The data obtained from the maximum amplitude in each treatment were analyzed using ANOVA followed by the Tukey–Kramer multiple com-parison test.

**Figure 4:**
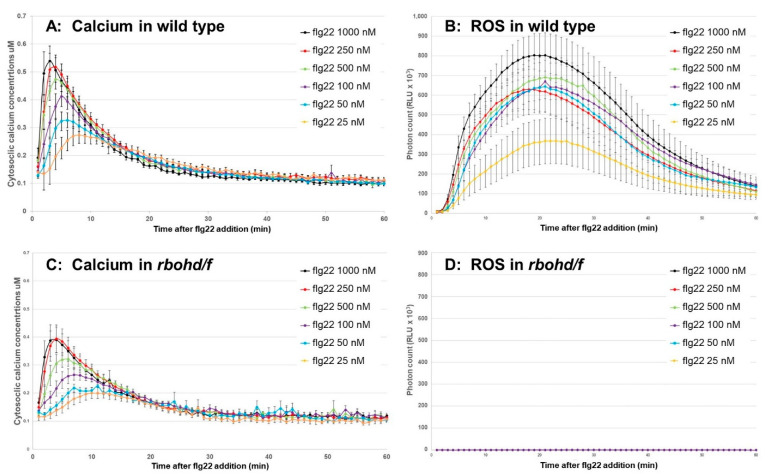
The effect of apoplastic ROS generated by RBOHs on calcium signaling. A flg22-dose-dependent response in cytosolic calcium elevation (**A**,**C**) and apoplastic ROS production (**B**,**D**) was compared between the wild-type plant (**A**,**B**) and the double knockout mutant *rbohd/f*, both of which are expressing apoaequorin. Measurements of calcium and ROS were performed over 60 min following addition of flg22 at a final concentration of 25, 50, 100, 250, 500, or 1000 µM.

To explore how calcium and ROS signals interact in PTI responses, the present study evaluated the effects of perturbing one signal on the other in *Arabidopsis* leaves upon flg22 addition. To this end, the flg22-induced calcium or ROS response was perturbed using the pharmacological agents and a genetic mutant, *rbohd/f*, shown in Figure 1, Figure 2, Figure 3 and Figure 4. Dynamic changes in cytosolic calcium and apoplastic ROS levels are presented as the timing of the maximum amplitude (C and D of Supplementary Figures S1–S11), the maximum amplitude (Figure 1 and Figure 3; E and F of Supplementary Figures S1–S11), the integrated values over 60 min (G and H of Supplementary Figures S1–S11), and the integrated values between 50–60 min (last 10 min of the kinetics measured) as the recovery of the second messenger (I and J of Supplementary Figures S1–S11). Detailed kinetic data are also shown (A and B of Supplementary Figures S1–S11).

### 2.1. Effects of Calcium Manipulation on Apoplastic ROS

#### 2.1.1. Effects of Nonspecific Calcium Channel Blockers on Apoplastic ROS Signaling

Lanthanum (La^3+^) and gadolinium (Gd^3+^) are nonspecific blockers of calcium channels [46]. As expected, when leaves were pretreated with La^3+^ or Gd^3+^, we observed a dose-dependent inhibition of the flg22-induced calcium response: a delay in the peak time and a decrease in the peak amplitude (Figure 1A,C), and a decrease in the total cytosolic calcium concentration in 60 min (Supplementary Figures S1 and S2). Under higher concentrations of lanthanum and gadolinium, flg22 induced only approximately half of the cytosolic calcium concentration that was observed without any inhibitor compounds (Supplementary Figures S1E and S2E). After flg22 treatment, the cytosolic calcium content of mock-treated plants had returned to baseline levels, whereas La^3+^-and Gd^3+^-treated plants had 145% of baseline, even at 60 min. (Supplementary Figures S1I and S2I). Overall, nonspecific calcium channel blockers significantly perturbed cytosolic calcium signatures by reducing the influx of calcium into the cytosol.

ROS induced by flg22 in leaf discs pre-treated with La^3+^ or Gd^3+^ was significantly slowed in the peak time and reduced in the amplitude (Figure 1B,D; Supplementary Figures S1 and S2). Notably, under higher concentrations of calcium channel blockers, the peak of ROS amplitude was reduced to <20% of non-treated control, which was a greater inhibition than that seen for cytosolic calcium (Supplementary Figures S1F and S2F). The recovery after the peak of flg22-induced ROS was also perturbed; more ROS remained at the lower concentrations of La^3+^ and Gd^3+^ (25–100 µM), whereas higher concentrations of the channel blockers (>100 µM) completely suppressed flg22-induced ROS production (Supplementary Figures S1J and S2J). These results indicate that general calcium channel functionality is required for appropriate flg22-induced ROS signatures.

#### 2.1.2. Effects of Specific Calcium Channel Blockers on Apoplastic ROS Signaling

The existence of voltage-dependent calcium channels (VDCCs) in plant cell plasma membranes has long been observed in plant electrophysiology [47]. Mibefradil (MB) is known to block both T-type (low-voltage activated family) and L-type (high-voltage activated family) VDCCs, while diltiazem (DI) blocks only L-type calcium channels in animals [48]. Verapamil (VP) has been reported to block HACC (hyperpolarization-activated calcium channel)-mediated calcium influx [49]. When treated with MB, DI and VP, flg22-induced calcium production was slightly diminished in a peak amplitude of at most 68–81% of that of the mock control (Figure 1C;Supplementary Figures S3E, S4E, and S5E). Interestingly, MB and DI significantly slowed the recovery of cytosolic calcium concentrations (Supplementary Figures S3I and S4I), whereas VP did not have similar effects on the recovery (Supplementary Figure S5I). Notably, these VDCC blockers exhibited marked inhibitory effects on flg22-induced ROS production. Peak amplitudes and integrated calcium concentrations elevated in 60 min were decreased in a dose-dependent manner and completely attenuated at high concentrations of the channel blockers, MB, DI, and VP (Figure 1D; Supplementary Figure S3F,H, S4F,H, and S5F,H). Together, these results suggest that VDCCs play a partial role for initiating calcium signaling but are required for generating a proper apoplastic ROS signal.

#### 2.1.3. Effects of Increased Cytosolic Calcium on Apoplastic ROS Signaling

To measure the direct effect of increased cytosolic calcium on apoplastic ROS signaling, we treated with a calcium ionophore, calcimycin, and then applied calcium (as CaCl_2_) instead of flg22. As shown in Figure 2 and Supplementary Figure S9, apoplastic ROS signal was increased depending on the amount of calcium ions applied. This result suggests that apoplastic ROS generators, perhaps RBOHs, are directly affected by intracellular calcium signals.

### 2.2. Effects of ROS Manipulation on Cytosolic Calcium

#### 2.2.1. Effects of Manipulation of ROS Signaling on Cytosolic Calcium Signaling

Next, we explored how ROS manipulations affect flg22-induced calcium signals. N-Acetyl cysteine (NAC) is an antioxidant that acts as an effective intracellular and extracellular ROS scavenger. As shown in Figure 3B,D, NAC suppressed flg22-induced ROS production in a dose-dependent manner, where NAC completely abolished the ROS signal at high concentrations, 500 µM–1 mM (Figure 3B,D; Figure S6).

For cytosolic calcium induced by flg22, NAC showed a slight inhibitory effect. At lower concentrations (25–250 µM), NAC lowered the peak amplitude, while at high concentrations, NAC lowered the amplitude even more so (Figure 3A,C; Supplementary Figure S6E). However, NAC did not abolish flg22-induced calcium as shown in flg22-induced ROS at the high concentrations, i.e., 500 µM–1 mM NAC (Figure 3A; Supplementary Figure S6E). These data suggest that a proper ROS signaling is not required for the initiation of the calcium signaling, but necessary to intensify the amplitude of cytosolic calcium levels at the appropriate time and for the recovery of cytosolic calcium upon flg22 addition.

#### 2.2.2. Effects of Manipulation of Apoplastic Specific ROS Signaling on Cytosolic Calcium Signaling

Catalase is an ROS scavenging enzyme that catalyzes the decomposition of ROS to water and oxygen. To examine the effects of decreased apoplastic ROS on flg22-induced calcium signals, plants were pretreated with various concentrations of catalase (5–100 units/µL). Catalase dramatically reduced flg22-induced apoplastic ROS production at all concentrations tested (Figure 3D; Supplementary Figure S7). In contrast, catalase showed only limited inhibition of flg22-induced calcium elevation (Figure 3C; Supplementary Figure S7). These results suggest that apoplastic ROS signal has a limited impact on cytosolic calcium signaling.

### 2.3. RBOHs Are Primarily Impacted by Calcium Signaling, but Has Limited Impact on Calcium Signaling

Diphenyleneiodonium (DPI) is a specific inhibitor of plasma membrane-bound NOXs and RBOHs that generate an apoplastic ROS burst and ROS-mediated immune responses. Consistent with previous reports [50], DPI inhibited flg22-induced ROS signatures in a dose-dependent manner (Figure 3D; Supplementary Figure S8). For the cytosolic calcium signal, DPI has only limited inhibitory effects (Figure 3C; Supplementary Figure S8), although it caused inhibition of the peak and reduction of the recovery to baseline at the high concentrations. These results suggest that apoplastic ROS signal produced by RBOHs has no significant impact on the initiation of the cytosolic calcium signal, but contributes to the amplitude of cytosolic calcium signal and its recovery to baseline.

To validate the results obtained using DPI, we next tested the influence of RBOH-dependent ROS on calcium signaling using a genetic material, which is a aequorin expressing transgenic lines in the double mutant background, *rbohd rbohf* [31]. As shown in Figure 4, flg22-induced ROS was completely attenuated in the *rbohd/f* mutant, while a dose-dependent response was observed in the wild-type plants (Figure 4B,D). In contrast, cytosolic calcium was still increased dependent on the dose of flg22 applied, although the intensity of the response was slightly weakened in the *rbohd/f* mutant in comparison to the wild type plants (Figure 4A,C; Supplementary Figures S10 and S11). These results suggests that initiation of cytosolic calcium elevation upon flg22 addition was independent of apoplastic ROS signal produced by RBOHs.

## 3. Discussion

In the present study, we evaluated the effect of perturbation of the calcium signaling on the ROS signaling, and vice versa. The results provided us with a better idea of how ROS and calcium signatures are required for proper dynamics for one another signature. As described below, we discuss how calcium and ROS signatures interact for the PTI response.

### 3.1. An Undisturbed Cytosolic Calcium Signal Is Required for Apoplastic ROS Signaling

The data examined in this study suggest that the flg22-induced calcium signal is required for the formation of subsequent apoplastic ROS signaling, as shown in Figure 1. Other data from experiments using calcimycin (Figure 2 and Supplementary Figure S9) and the *rbohd/f* mutant (Figure 4 and Supplementary Figure S11) suggest that RBOHs are a hub to form a calcium-dependent ROS signal, as proposed in the previous literature [4]. It is likely that RBOHs are inactive at low cytosolic calcium levels, due to a necessity for calcium-dependent phosphorylation by CPKs and conformational changes with calcium binding on EF-hand motifs on the N-terminal extension of RBOHs [35,36]. If that threshold is unreached, or even if only a few RBOH proteins are activated, then it appears that the plant cannot initiate an apoplastic ROS signal. This can be predicted to have a profound effect on signal transmission from cell to cell, as apoplastic ROS signal has been shown to be necessary for plant immune responses such as stomatal closure and the hypersensitive response. The peak timing of the flg22-induced ROS response, as shown in Supplementary Figures S1 and S2, also slowed down by almost 10–15 min in a dose-dependent manner under La^3+^ and Gd^3+^ treatments. This delay seemed synchronized with the calcium response, suggesting that subsequent activation of RBOHs is perturbed due to disruption of calcium signaling. These data demonstrated that cytosolic calcium elevation upon flg22 addition is the initial signal to orchestrate ROS signaling.

### 3.2. ROS Signal Partially Regulates Calcium Signaling

The results from the experiments using the ROS scavengers NAC and catalase and the *rbohd/f* mutant (Figure 3 and Figure 4) suggest that proper ROS signal is important for forming a sufficient calcium signaling but not for initiating calcium signatures. Catalase scavenges ROS in the apoplast and lowers ROS levels by nearly 100% at all concentrations used, which verifies its use as an effective ROS scavenger (Figure 3 and Supplementary Figure S7). Similar to NAC, catalase slightly diminished the flg22-induced calcium signature but did not affect the timing of the maximum amplitude to any observed extent. Together, these results suggest a limited effect of apoplastic and cytosolic ROS in regulating flg22-induced calcium signaling dynamics. Considering that catalase cannot enter the plasma membrane, it is likely that these effects were solely mediated by eliminating apoplastic ROS, as seen in the *rbohd/f* mutants, in which flg22 still induced calcium signaling with a small negative effect (Figure 3 and Supplementary Figure S7). We replicated this observation by inhibiting RBOHs using DPI (Figure 3 and Supplementary Figure S8).

Several pathways have been suggested to be involved in ROS-dependent regulation of calcium signaling. Calcium signaling-related channels such annexins, CNGCs, and stellar potassium outward rectifier (SKOR) have been shown to be activated in high redox states, perhaps due to pH changes or H_2_O_2_ directly [44,51]. Calcium channels may require cytosolic ROS to be activated and relieve calcium levels from the cytosol, perhaps via S-nitrosylation or other redox-dependent activation. Very recently, HPCA1 was identified as a cell surface H_2_O_2_ receptor that was reported to directly activate a calcium channel upon ligand recognition [52]. This could be an ROS-induced calcium signaling, although HPCA1 is not required for MAMP-induced calcium responses,

The cytosolic calcium levels in the presence of high concentrations of NAC (500 µM–1 mM) showed that the cells were unable to reduce the flg22-induced cytosolic calcium to baseline levels (Figure 3 and Supplementary Figure S6). It appears that NAC affects the flg22-induced calcium signaling, which is perhaps due to its cytosolic ROS scavenging properties, as the apoplastic ROS scavenger catalase did not have any impact on flg22-induced calcium signaling recovery, suggesting that cytosolic ROS plays a more important role in governing calcium dynamics, namely, recovery. Predicted VDCC channels such as CNGC2 and CNGC4 have been shown to have autoimmune phenotypes in its knockout mutants [30]. This autoimmunity is likely in part due to the inability of such plants to regulate calcium recovery properly. Interestingly, the VDCC inhibitors caused similar recovery perturbations and kept the calcium signaling from ending. This implies that undiscovered VDCC channels may be responsible for engaging with efflux channels such as ACAs and CAXs. It would be of interest to examine ACAs and CAXs to see if redox or pH affects them as well as VDCCs, as they may require proper ROS levels in the cell to form correct cytosolic calcium signaling.

### 3.3. Conclusions

**Figure 5:**
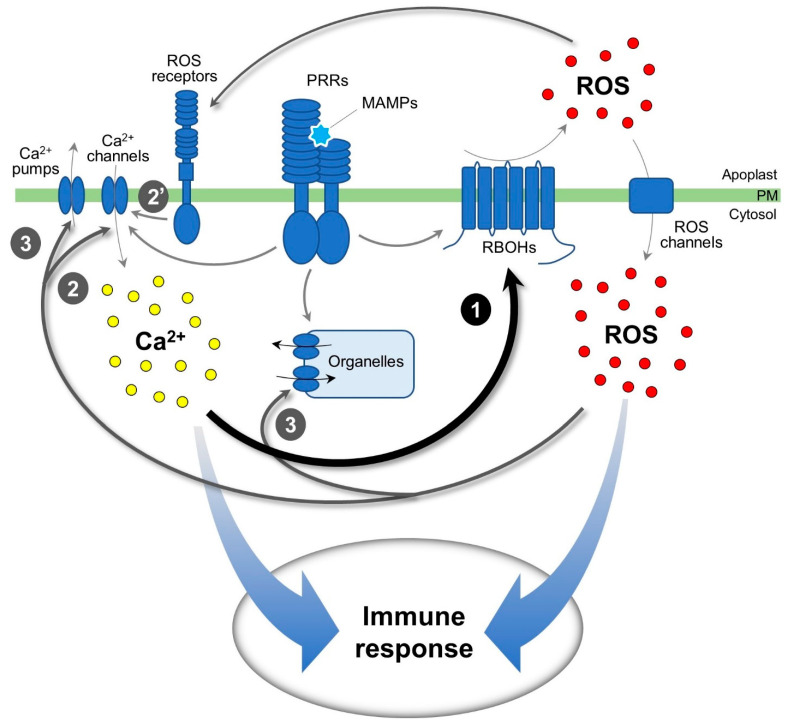
Mutual interplay of calcium and ROS signaling in plant immune response. The MAMP-induced calcium signal has a significant impact on the initiation and amplification of the apoplastic ROS signal, where RBOHs are a central hub for the calcium-dependent ROS signaling (1). In contrast, The MAMP-induced ROS signal has a partial effect on calcium signal but is required for substantial calcium signaling, where a proper formation of calcium signature relies on intracellular ROS-dependent regulation of calcium channels (2) and extracellular ROS-dependent regulation of calcium channels via ROS receptors (2′). In addition, ROS is likely involved in the recovery of cytosolic calcium concentration via regulating calcium pumps (3). The calcium and ROS signals orchestrate together at the proper time and amplitude of pattern-triggered immune response.

In the present study, our data demonstrated that inhibition of the flg22-induced calcium signature has a significant impact on the initiation and amplification of the ROS signaling (Figure 5). In this case, RBOHs are a central hub for the interplay during calcium-dependent ROS signaling. In contrast, inhibition of ROS signatures has a lesser effect on calcium signaling but is important for forming substantial amplitude of cytosolic calcium elevation. It appears that a proper rise in cytosolic calcium concentrations relies on ROS signaling that regulates calcium channels directly or indirectly via ROS receptors, which could be a homologous protein of HPCA1 (Figure 5). Otherwise, ROS is likely involved in the recovery of cytosolic calcium concentration by regulating calcium pumps (Figure 5). In conclusion, calcium signal plays an essential role for generating ROS signaling where it is required for both initiation and tuning of ROS signaling, whereas ROS signal is required for the tuning of, but not the initiation of, calcium signaling. Our data verify the mutual interplay between calcium and ROS signals, where the cytosolic calcium threshold to activate RbohD and other ROS machinery is quite specific for flg22-induced response and may be different from responses to other MAMPs and DAMPs. Indeed, there are notable mechanistic differences during dynamic changes in cytosolic calcium evoked by flg22, elf18, and Pep3 [53]. Given that fact, examining other MAMPs, such as chitin or elf18, and DAMPs, such as Peps and extracellular ATP, to see if their ROS and calcium regulation dynamics differ would provide insight into detailed mechanisms in the interplay of calcium and ROS signals.

## 4. Materials and Methods

### 4.1. Plants

All plants used in this study were Columbia-0 (Col-0) *Arabidopsis* expressing apoaequorin for cytosolic calcium detection [42,45]. The aequorin enzyme oxidizes coelenterazine (CTZ) in the presence of calcium ions, where blue light (469 nm) is emitted. The *rbohd/f* mutant [34] expressing apoaequorin was made through cross pollination. For plant growth, sterile *Arabidopsis* seeds were plated on MS medium containing 1% sucrose and 0.5% Phytagel agar. Plated seeds were wrapped in aluminum foil and vernalized at 4 °C for 3 days to synchronize germination. Plates were then grown vertically in a growth chamber (Conviron) at 22 °C, light intensity 120 μE/m^2^/s for 5 days, and then plants were transplanted to soil and grown at 22 °C, 110 μE/m^2^/s for 4 weeks in 16-h light conditions, fertilized once a week with 200 ppm of water-soluble all-purpose nutrients (Miracle-Gro).

### 4.2. Chemicals

Unless mentioned otherwise, all reagents were obtained from Sigma-Aldrich and dissolved in water at a concentration of 100 mM and then diluted to 25, 50, 100, 250, 500, and 1000 µM for individual experiments. Exceptions are noted under the descriptions below. Lanthanum (III) chloride (LaCl_3_) and gadolinium (III) chloride (GdCl_3_) were obtained from Beantown Chemical. Mibefradil, verapamil, N-acetyl cysteine and nifedipine were obtained from Sigma Aldrich and dissolved in DMSO. Final DMSO concentration was less than 0.01%. Diltiazem was obtained from TCI. Catalase from bovine liver was also obtained from Sigma Aldrich and dissolved in water followed by dilution to 1, 10 and 100 units/µL. Calcimycin A23187 was obtained from Sigma Aldrich and diluted to 100 µM for use with calcium concentrations of 10, 100, and 1000 µM.

### 4.3. Cytosolic Free Calcium Measurement

Leaf discs (3-mm diameter) were prepared from rosette leaves of 6-week-old plants and were transplanted to 96-well plates containing 50 μL of a reconstitution buffer including 20 µM CTZ, 10 mM CaCl_2_, and 2 mM MES (pH 5.7). Plates were organized by eight replicates for each treatment or genotype, and experiments were performed at least three times (*n* = 24 in total). Once wells were set up, the plates were wrapped in tin foil, placed in a tin box for light shielding and incubated overnight to allow CTZ to saturate the plant cells. For calcium measurement, leaf discs were treated with calcium or ROS pharmacological agents for 30 min and then treated with 1 μM flg22 elicitor. The luminescence signals were then measured using a luminometer (Promega GloMax Navigator) set to take readings every 1 sec for 60 min. After the measurement, a discharging solution, including 2 M CaCl2 and 20% (*v*/*v*) ethanol, was added to estimate all residual apoaequorin in the cells. Cytosolic free calcium concentrations were calculated as described previously [45,51].

### 4.4. Apoplastic ROS Measurement

A luminol-based chemiluminescence assay was performed as described previously [54,55]. Briefly, leaf discs (3-mm diameter) from rosette leaves of 6-week-old plants were transferred to 96-well microplates containing 50 µL of deionized water overnight at 22 °C. Plates were organized by eight replicates for each treatment and were performed at least three times (*n* = 24) for each treatment examined. Water was carefully removed from each well via a multichannel pipet to ensure no damage to the leaf discs, and then calcium or ROS pharmacological agents were added. After 30 min of treatment, the leaf discs were washed three times with deionized water, and then 0.2 mM luminol derivative (L-012) and 20 mg/L HRP were added together with 1 µM of flg22 elicitor. The luminescence signals were then measured using a luminometer (Promega GloMax Navigator) set to take readings every 1 s for 60 min. The results were expressed as relative light units (RLUs per leaf disc) after subtraction of the data at time 0 from those at each time point of the measurement.

### 4.5. Data Analysis

Three experiments of eight samples (*n* = 24) for each treatment were averaged together for data analysis. Statistical analysis was performed using Student’s t-test compared to nontreated samples.

## Data Availability

Data is contained within the article.

## Supplementary Materials

The following are available online at https://www.mdpi.com/article/10.3390/plants11010014/s1, Figure S1: Effects of La^3+^ on flg22-induced cytosolic calcium signaling and apoplastic ROS signaling, Figure S2: Effects of Gd^3+^ on flg22-induced cytosolic calcium signaling and apoplastic ROS signaling, Figure S3: Effects of mibefradil on flg22-induced cytosolic calcium signaling and apoplastic ROS signaling, Figure S4: Effects of diltiazem on flg22-induced cytosolic calcium signaling and apoplastic ROS signaling, Figure S5: Effects of verapamil on flg22-induced cytosolic calcium signaling and apoplastic ROS signaling, Figure S6: Effects of NAC on flg22-induced cytosolic calcium signaling and apoplastic ROS signaling, Figure S7: Effects of catalase on flg22-induced cytosolic calcium signaling and apoplastic ROS signaling, Figure S8: Effects of DPI on flg22-induced cytosolic calcium signaling and apoplastic ROS signaling, Figure S9: Effects of increased cytosolic calcium on flg22-induced cytosolic calcium signaling and apoplastic ROS signaling, Figure S10: Effects of different concentrations of flg22 on cytosolic calcium elevation and apoplastic ROS production in wild type, Figure S11: Effects of different concentrations of flg22 on cytosolic calcium elevation and apoplastic ROS production in a double knockout mutant, *rbohd/f*.
