# Supplementary material for: Crosstalk between Calcium and ROS Signaling during Flg22-Triggered Immune Response in Arabidopsis Leaves
Source: Plants (Basel). 2021 Dec 21;11(1):14. doi: 10.3390/plants11010014 (PMC8747291; doi:10.3390/plants11010014)
Supplement: Supplementary file 1 [file plants-11-00014-s001.zip › plants-1442836-supplementary.pdf]

plants

Crosstalk between calcium and ROS signaling during flg22-triggered immune response in Arabidopsis leaves

Matthew J. Marcec[1,2] and Kiwamu Tanaka[1,2,*]

[1] Department of Plant Pathology, Washington State University, Pullman, WA 99164, USA
[2] Molecular Plant Sciences Program, Washington State University, Pullman, WA 99164, USA
* Correspondence: kiwamu.tanaka@wsu.edu

Supplementary Materials

Figure S1: Effects of La^{3+} on flg22-induced cytosolic calcium signaling and apoplastic ROS signaling.

Figure S2: Effects of Gd^{3+} on flg22-induced cytosolic calcium signaling and apoplastic ROS signaling.

Figure S3: Effects of mibefradil on flg22-induced cytosolic calcium signaling and apoplastic ROS signaling.

Figure S4: Effects of diltiazem on flg22-induced cytosolic calcium signaling and apoplastic ROS signaling.

Figure S5: Effects of verapamil on flg22-induced cytosolic calcium signaling and apoplastic ROS signaling.

Figure S6: Effects of NAC on flg22-induced cytosolic calcium signaling and apoplastic ROS signaling.

Figure S7: Effects of catalase on flg22-induced cytosolic calcium signaling and apoplastic ROS signaling.

Figure S8: Effects of DPI on flg22-induced cytosolic calcium signaling and apoplastic ROS signaling.

Figure S9: Effects of increased cytosolic calcium on flg22-induced cytosolic calcium signaling and apoplastic ROS signaling.

Figure S10: Effects of different concentrations of flg22 on cytosolic calcium elevation and apoplastic ROS production in wild type.

Figure S11: Effects of different concentrations of flg22 on cytosolic calcium elevation and apoplastic ROS production in a double knockout mutant, *rbohd/f*.

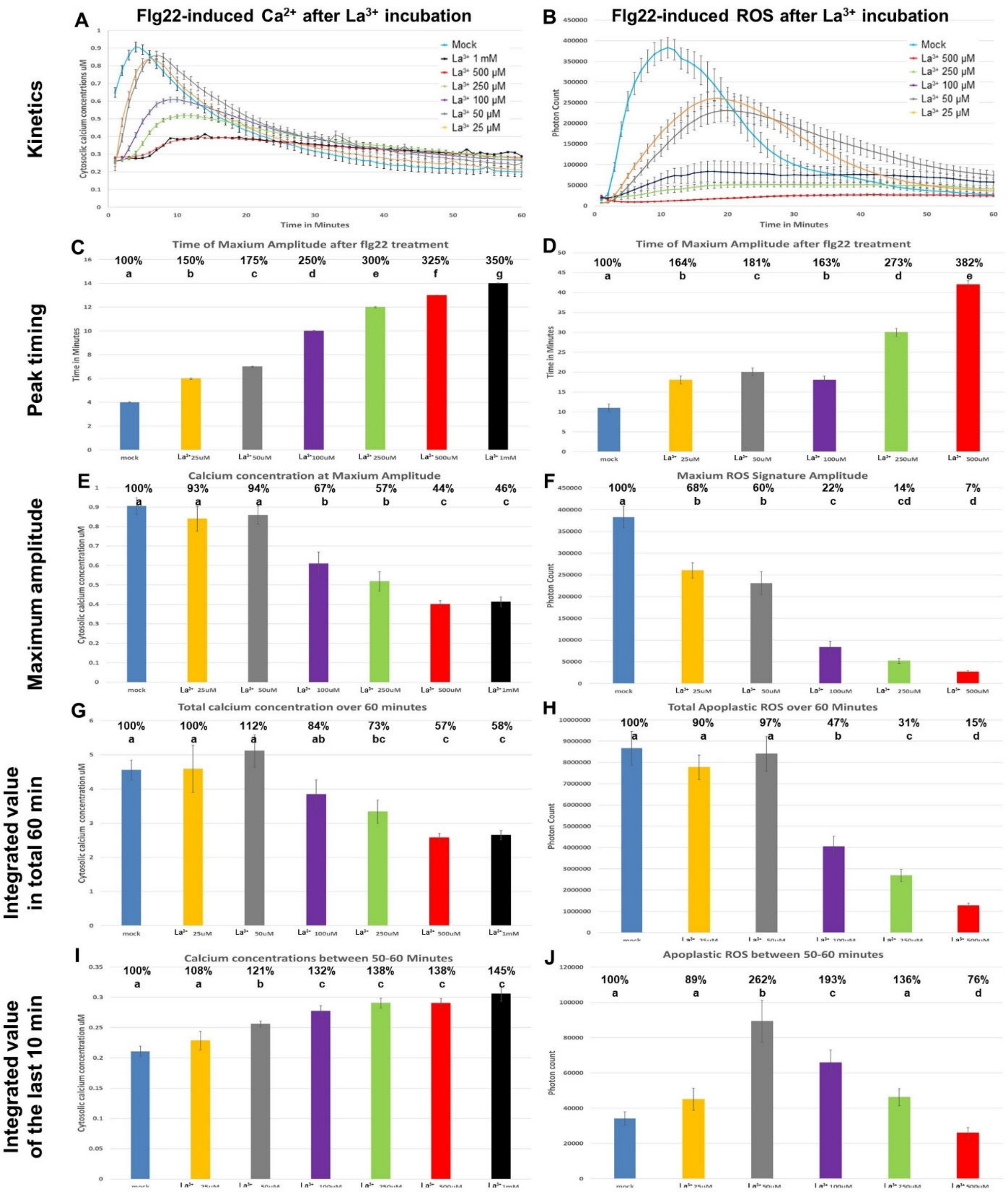

Supplementary Figure S1. Effects of La^{3+} on flg22-induced cytosolic calcium signaling and apoplastic ROS signaling. Line graphs show dynamic changes in cytosolic calcium elevation and apoplastic ROS production over 60 min after flg22 addition with or without pretreatment with La^{3+} (A and B). Histograms represent detailed data as the timing of the maximum amplitude (C and D), the maximum amplitude (E and F), the integrated values over 60 min (G and H), and the integrated values between 50–60 min (last 10 min of the kinetics measured) as the recovery of the second messenger (I and J). Leaf discs (n = 24) were preincubated for 30 min with La^{3+} (25 μM-1 mM) prior to 1 μM flg22 addition. Histogram values above bars represent percentage compared to non-treated plants. Different letters indicate statistically significant differences at $P < 0.05$ with Tukey-Kramer multiple comparison test.

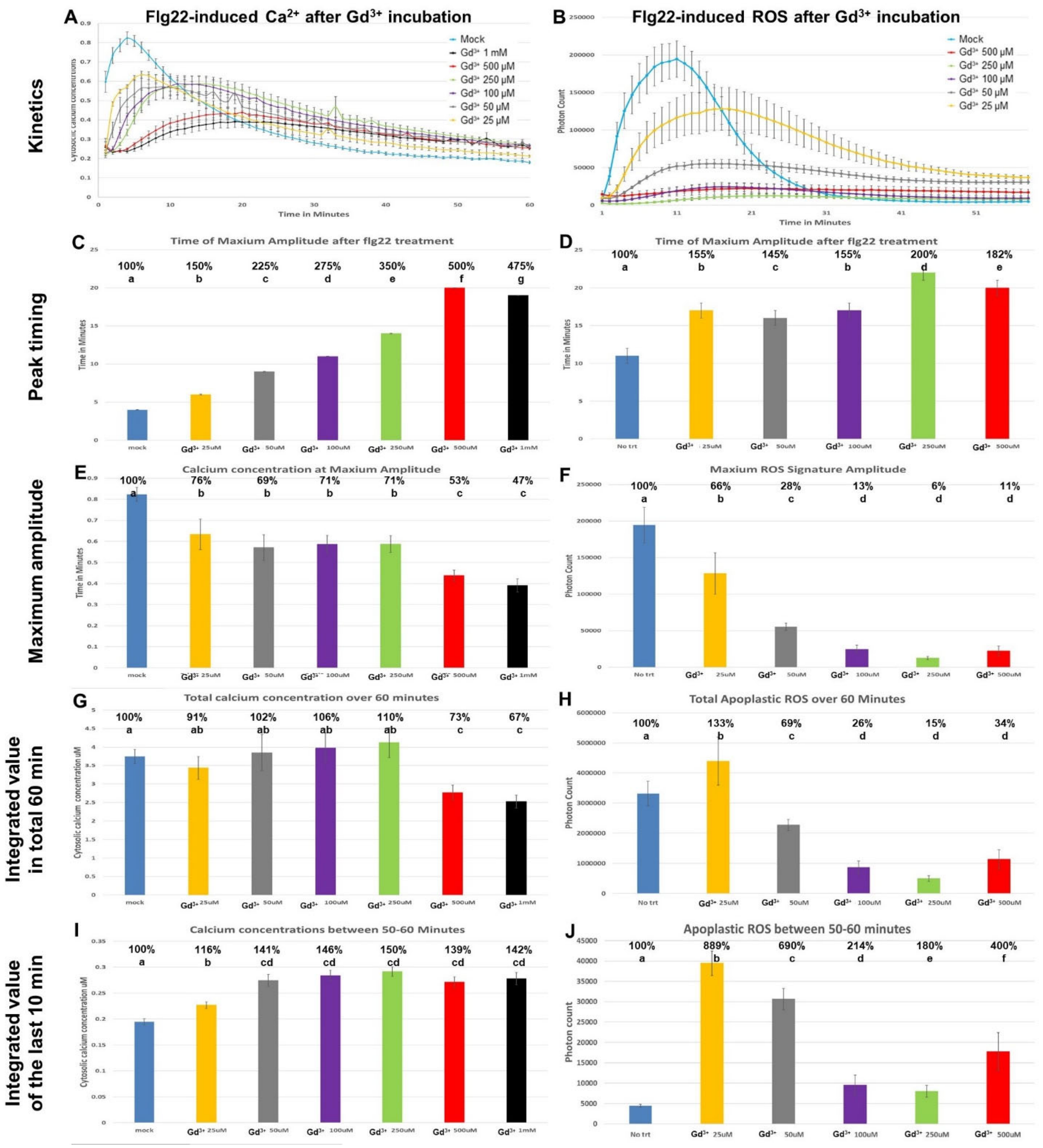

Supplementary Figure S2. Effects of Gd^{3+} on flg22-induced cytosolic calcium signaling and apoplastic ROS signaling. Line graphs show dynamic changes in cytosolic calcium elevation and apoplastic ROS production over 60 min after flg22 addition with or without pretreatment with Gd^{3+} (A and B). Histograms represent detailed data as the timing of the maximum amplitude (C and D), the maximum amplitude (E and F), the integrated values over 60 min (G and H), and the integrated values between 50–60 min (last 10 min of the kinetics measured) as the recovery of the second messenger (I and J). Leaf discs (n = 24) were preincubated for 30 min with Gd^{3+} (25 μM-1 mM) prior to 1 μM flg22 addition. Histogram values above bars represent percentage compared to non-treated plants. Different letters indicate statistically significant differences at $P < 0.05$ with Tukey-Kramer multiple comparison test.

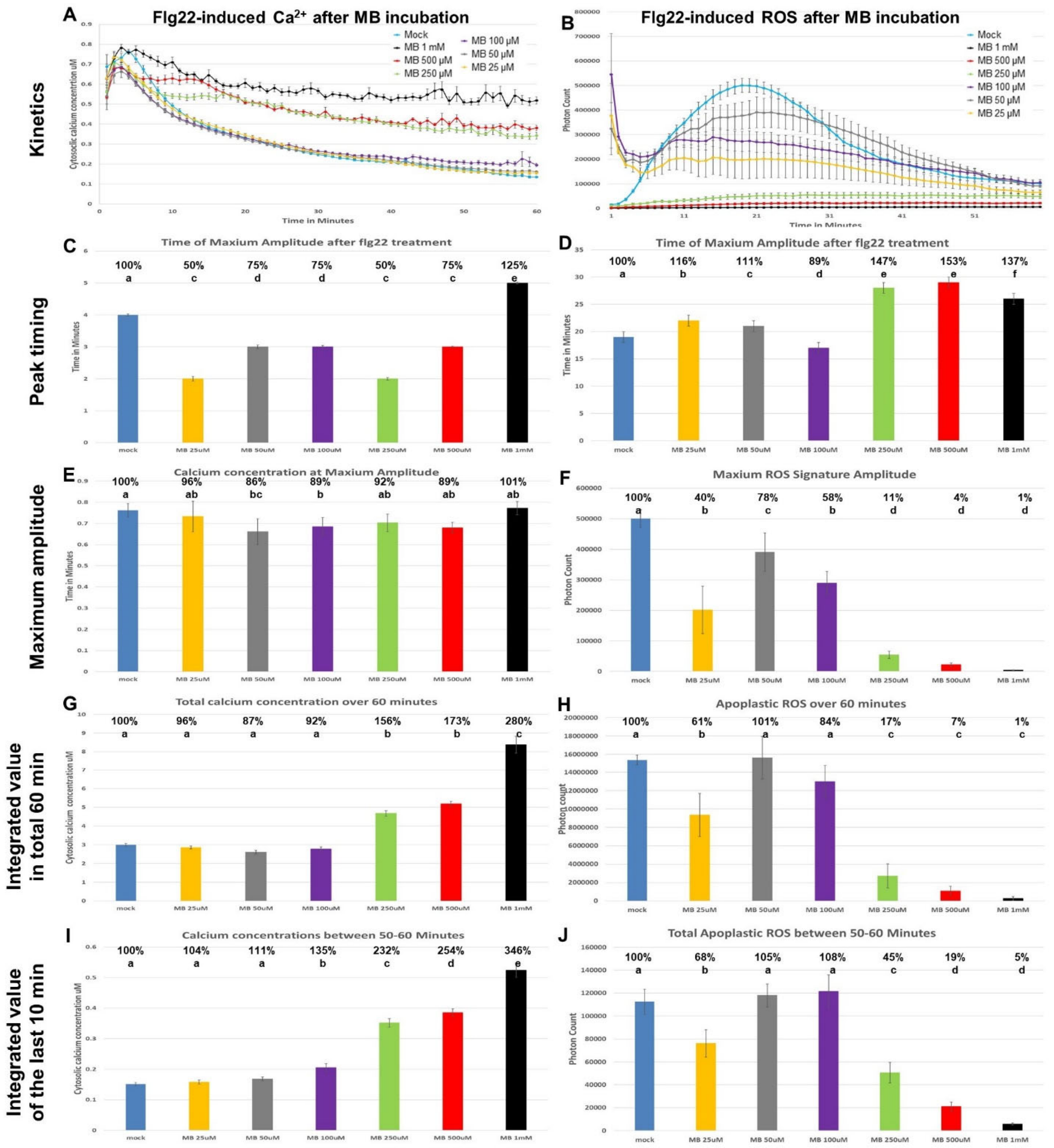

Supplementary Figure S3. Effects of mibefradil on flg22-induced cytosolic calcium signaling and apoplastic ROS signaling. Line graphs show dynamic changes in cytosolic calcium elevation and apoplastic ROS production over 60 min after flg22 addition with or without pretreatment with mibefradil or MB (A and B). Histograms represent detailed data as the timing of the maximum amplitude (C and D), the maximum amplitude (E and F), the integrated values over 60 min (G and H), and the integrated values between 50–60 min (last 10 min of the kinetics measured) as the recovery of the second messenger (I and J). Leaf discs (n = 24) were preincubated for 30 min with MB (25 μM-1 mM) prior to 1 μM flg22 addition. Histogram values above bars represent percentage compared to non-treated plants. Different letters indicate statistically significant differences at $P < 0.05$ with Tukey-Kramer multiple comparison test.

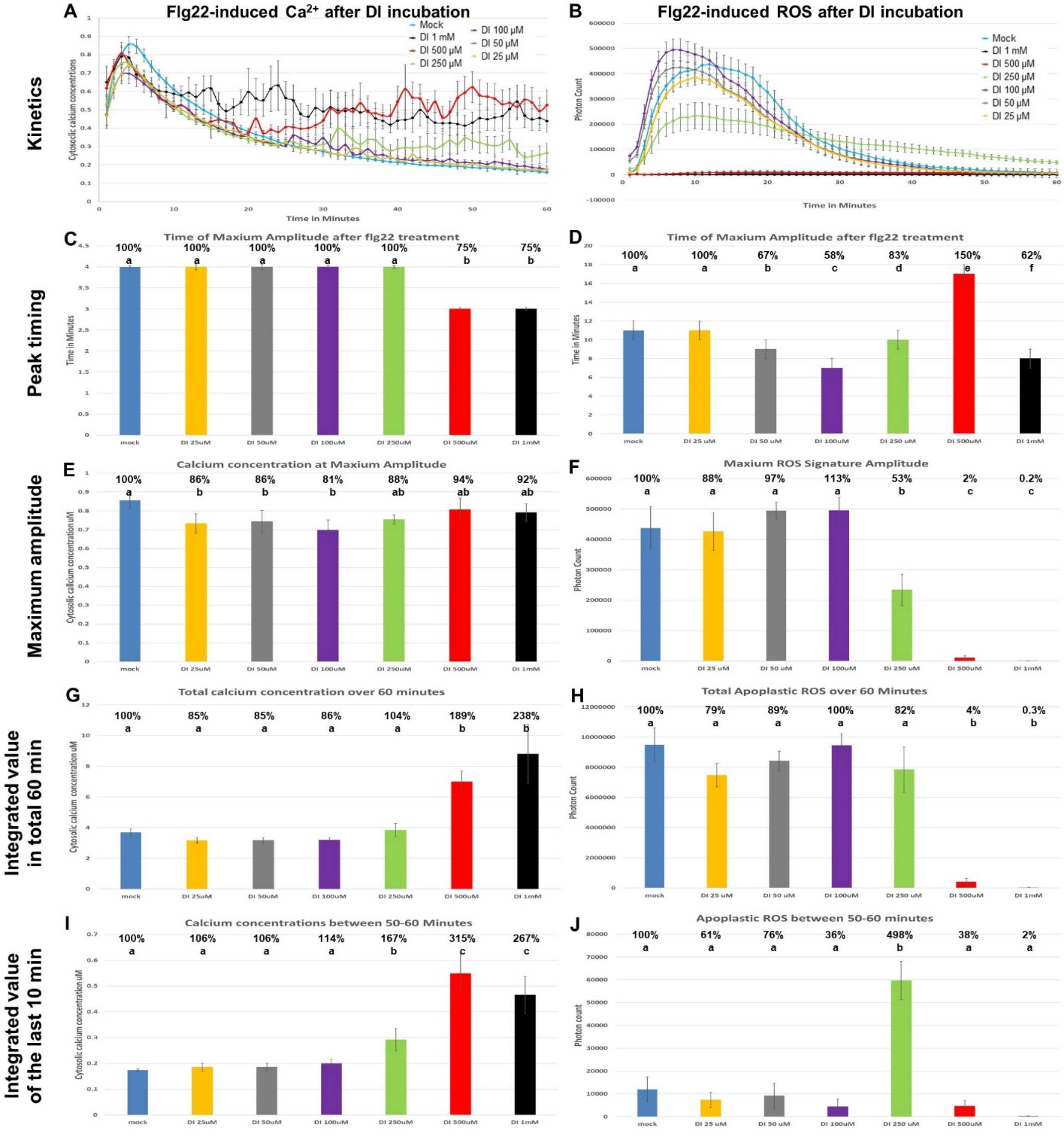

Supplementary Figure S4. Effects of diltiazem on flg22-induced cytosolic calcium signaling and apoplastic ROS signaling. Line graphs show dynamic changes in cytosolic calcium elevation and apoplastic ROS production over 60 min after flg22 addition with or without pretreatment with diltiazem or DI (A and B). Histograms represent detailed data as the timing of the maximum amplitude (C and D), the maximum amplitude (E and F), the integrated values over 60 min (G and H), and the integrated values between 50–60 min (last 10 min of the kinetics measured) as the recovery of the second messenger (I and J). Leaf discs (n = 24) were preincubated for 30 min with DI (25 μM-1 mM) prior to 1 μM flg22 addition. Histogram values above bars represent percentage compared to non-treated plants. Different letters indicate statistically significant differences at $P < 0.05$ with Tukey-Kramer multiple comparison test.

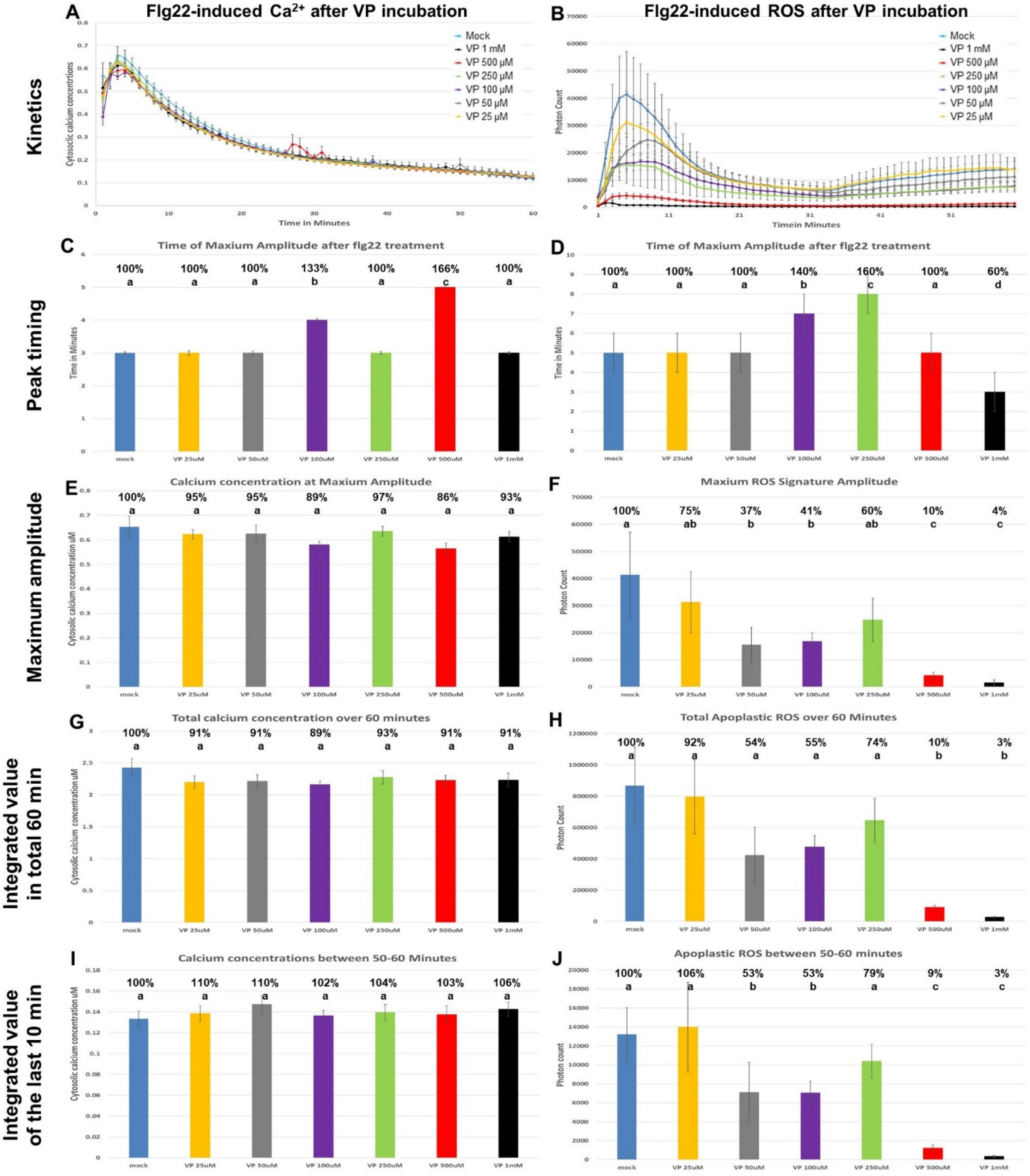

Supplementary Figure S5. Effects of verapamil on flg22-induced cytosolic calcium signaling and apoplastic ROS signaling. Line graphs show dynamic changes in cytosolic calcium elevation and apoplastic ROS production over 60 min after flg22 addition with or without pretreatment with verapamil or VP (A and B). Histograms represent detailed data as the timing of the maximum amplitude (C and D), the maximum amplitude (E and F), the integrated values over 60 min (G and H), and the integrated values between 50–60 min (last 10 min of the kinetics measured) as the recovery of the second messenger (I and J). Leaf discs (n = 24) were preincubated for 30 min with VP (25 μM-1 mM) prior to 1 μM flg22 addition. Histogram values above bars represent percentage compared to non-treated plants. Different letters indicate statistically significant differences at $P < 0.05$ with Tukey-Kramer multiple comparison test.

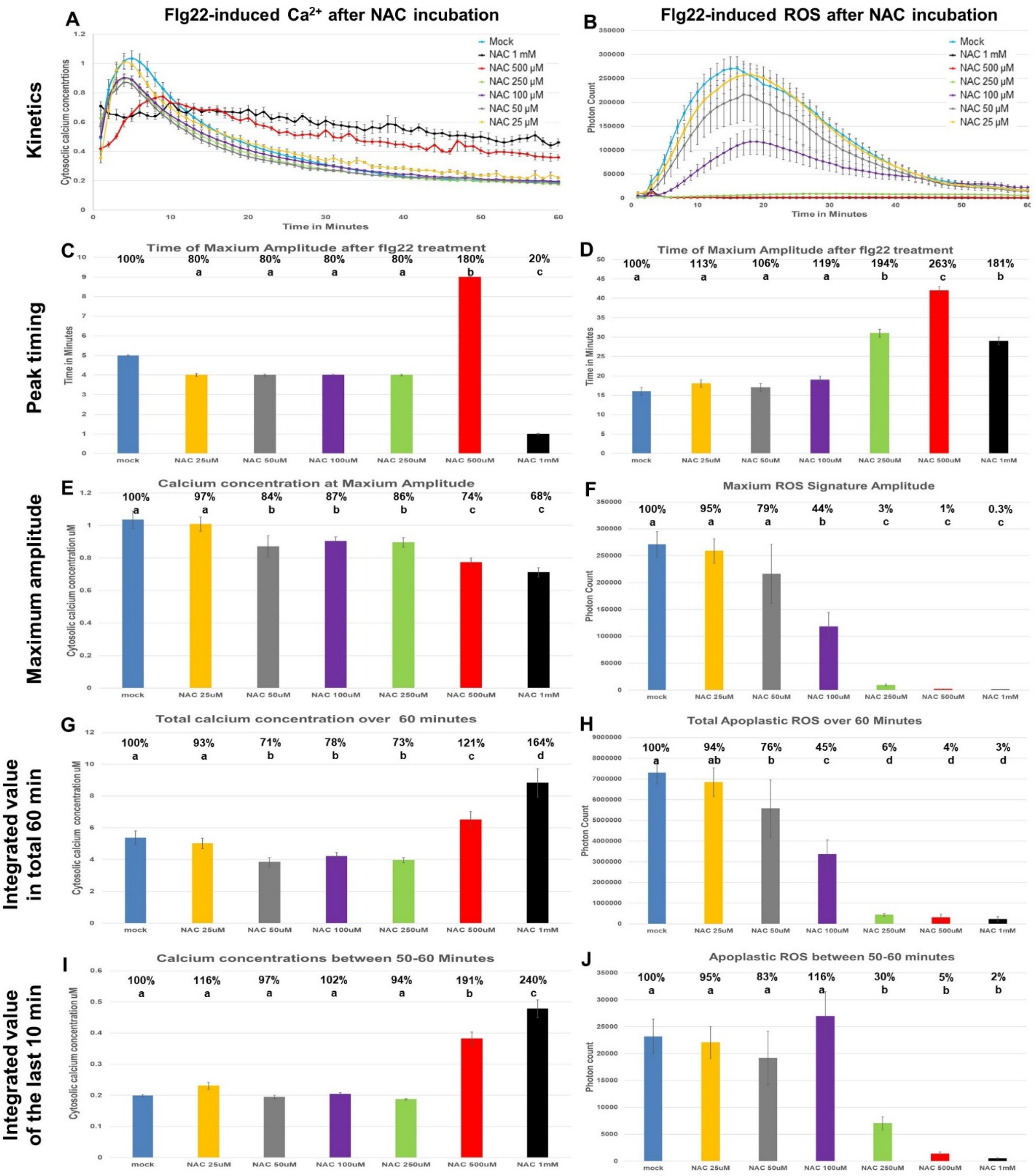

Supplementary Figure S6. Effects of NAC on flg22-induced cytosolic calcium signaling and apoplastic ROS signaling. Line graphs show dynamic changes in cytosolic calcium elevation and apoplastic ROS production over 60 min after flg22 addition with or without pretreatment with NAC (A and B). Histograms represent detailed data as the timing of the maximum amplitude (C and D), the maximum amplitude (E and F), the integrated values over 60 min (G and H), and the integrated values between 50–60 min (last 10 min of the kinetics measured) as the recovery of the second messenger (I and J). Leaf discs (n = 24) were preincubated for 30 min with NAC (25 μM-1 mM) prior to 1 μM flg22 addition. Histogram values above bars represent percentage compared to non-treated plants. Different letters indicate statistically significant differences at $P < 0.05$ with Tukey-Kramer multiple comparison test.

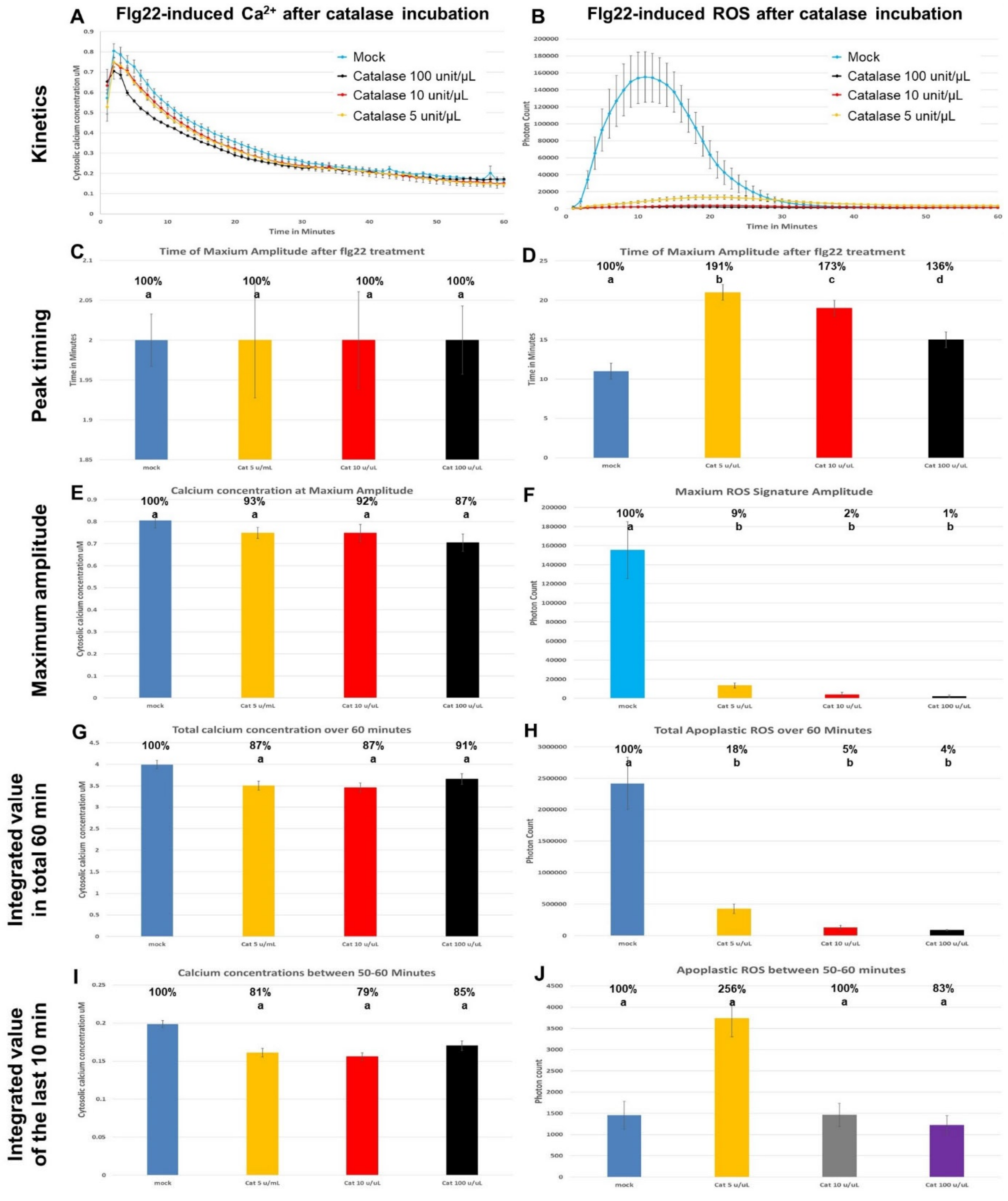

Supplementary Figure S7. Effects of catalase on flg22-induced cytosolic calcium signaling and apoplastic ROS signaling. Line graphs show dynamic changes in cytosolic calcium elevation and apoplastic ROS production over 60 min after flg22 addition with or without pretreatment with catalase (A and B). Histograms represent detailed data as the timing of the maximum amplitude (C and D), the maximum amplitude (E and F), the integrated values over 60 min (G and H), and the integrated values between 50–60 min (last 10 min of the kinetics measured) as the recovery of the second messenger (I and J). Leaf discs (n = 24) were preincubated for 30 min with catalase (5-100 units/μL) prior to 1 μM flg22 addition. Histogram values above bars represent percentage compared to non-treated plants. Different letters indicate statistically significant differences at $P < 0.05$ with Tukey-Kramer multiple comparison test.

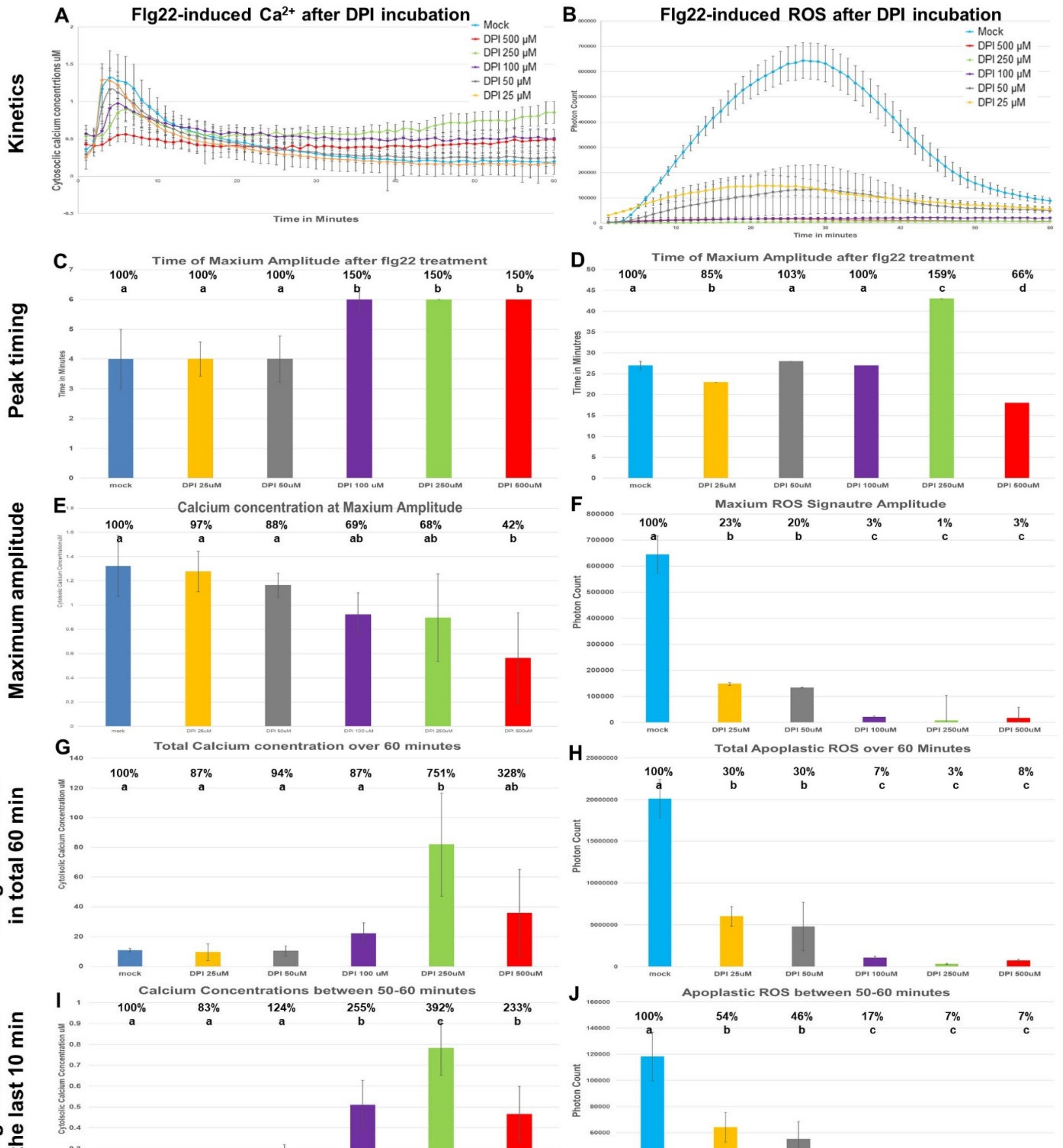

Supplementary Figure S8. Effects of DPI on flg22-induced cytosolic calcium signaling and apoplastic ROS signaling. Line graphs show dynamic changes in cytosolic calcium elevation and apoplastic ROS production over 60 min after flg22 addition with or without pretreatment with DPI (A and B). Histograms represent detailed data as the timing of the maximum amplitude (C and D), the maximum amplitude (E and F), the integrated values over 60 min (G and H), and the integrated values between 50–60 min (last 10 min of the kinetics measured) as the recovery of the second messenger (I and J). Leaf discs (n = 24) were preincubated for 30 min with DPI (25-500 μM) prior to 1 μM flg22 addition. Histogram values above bars represent percentage compared to non-treated plants. Different letters indicate statistically significant differences at $P < 0.05$ with Tukey-Kramer multiple comparison test.

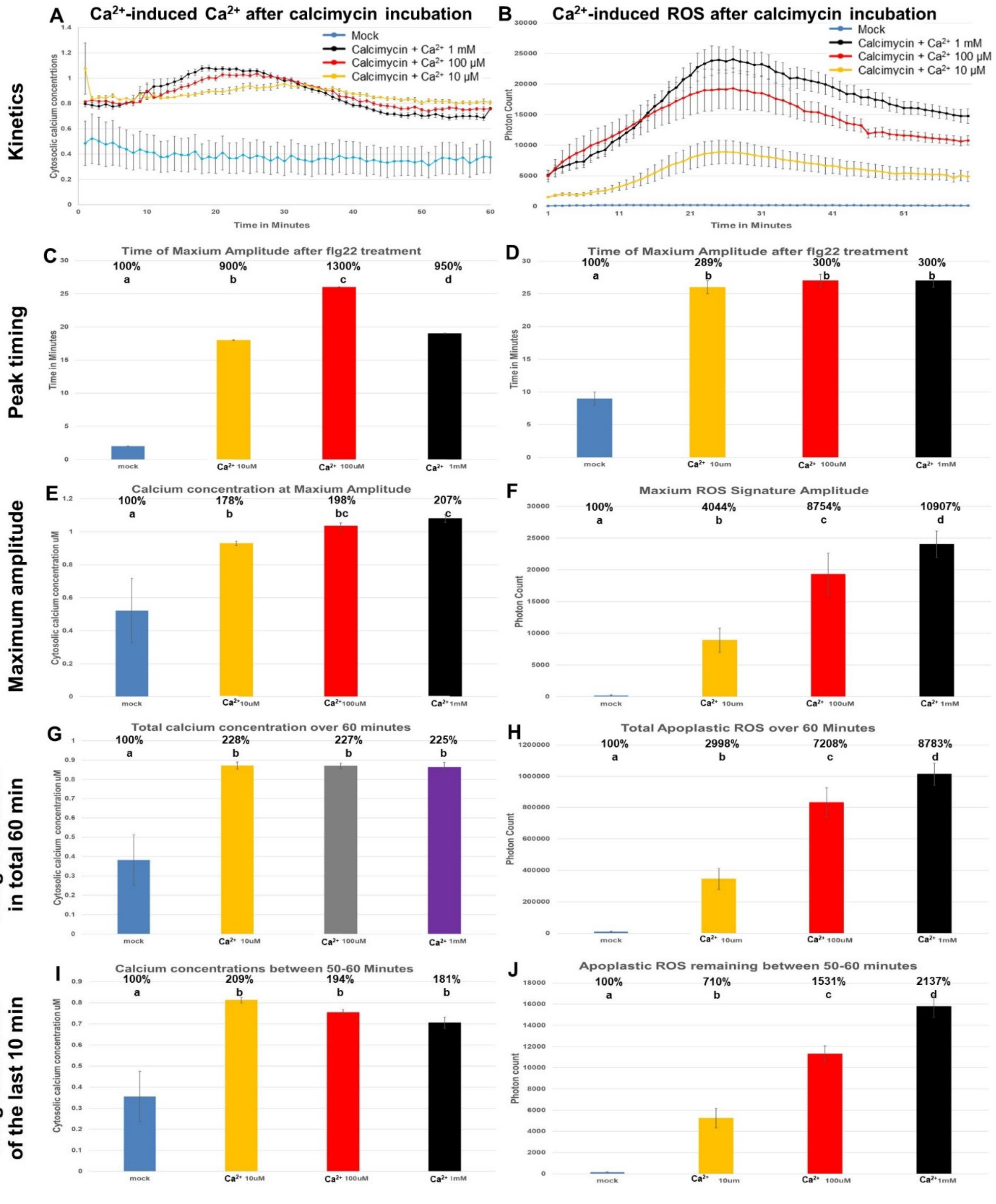

Supplementary Figure S9. Effects of increased cytosolic calcium on flg22-induced cytosolic calcium signaling and apoplastic ROS signaling. Line graphs show dynamic changes in cytosolic calcium elevation and apoplastic ROS production over 60 min after $CaCl_2$ addition with or without pretreatment with calcimycin (A and B). Histograms represent detailed data as the timing of the maximum amplitude (C and D), the maximum amplitude (E and F), the integrated values over 60 min (G and H), and the integrated values between 50–60 min (last 10 min of the kinetics measured) as the recovery of the second messenger (I and J). Leaf discs (n = 24) were preincubated for 30 min with calcimycin (100 μM) prior to addition of $CaCl_2$ (10-1000 μM). Histogram values above bars represent percentage compared to non-treated plants. Different letters indicate statistically significant differences at $P < 0.05$ with Tukey-Kramer multiple comparison test.

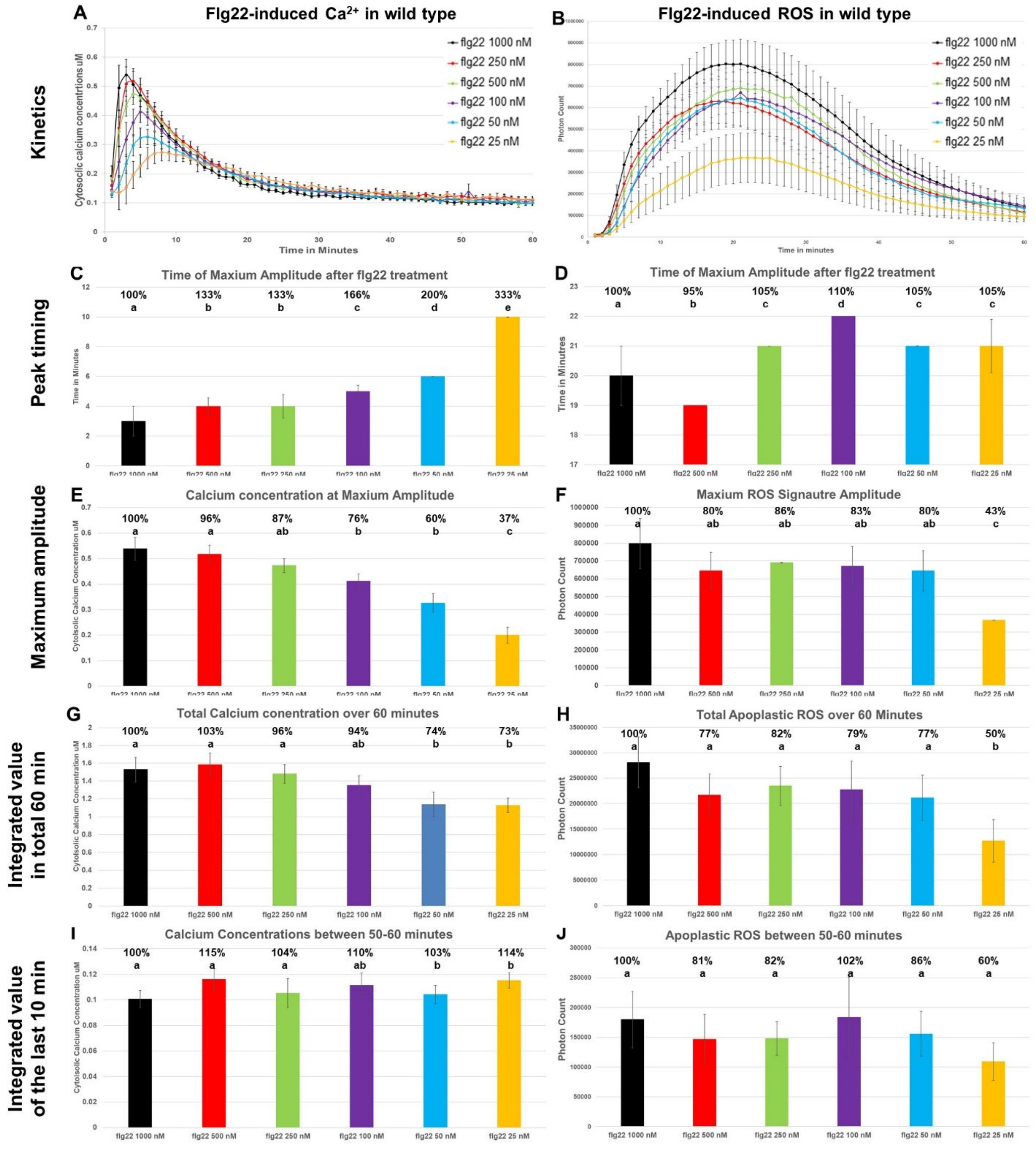

Supplementary Figure S10. Effects of different concentrations of flg22 on cytosolic calcium elevation and apoplastic ROS production in wild type. Line graphs show dynamic changes in cytosolic calcium elevation and apoplastic ROS production over 60 min after flg22 addition (A and B). Histograms represent detailed data as the timing of the maximum amplitude (C and D), the maximum amplitude (E and F), the integrated values over 60 min (G and H), and the integrated values between 50–60 min (last 10 min of the kinetics measured) as the recovery of the second messenger (I and J). Leaf discs (n = 24) were treated by flg22 addition at a final concentration of 25, 50, 100, 250, 500, or 1,000 μM. Histogram values above bars represent percentage compared to non-treated plants. Different letters indicate statistically significant differences at $P < 0.05$ with Tukey-Kramer multiple comparison test.

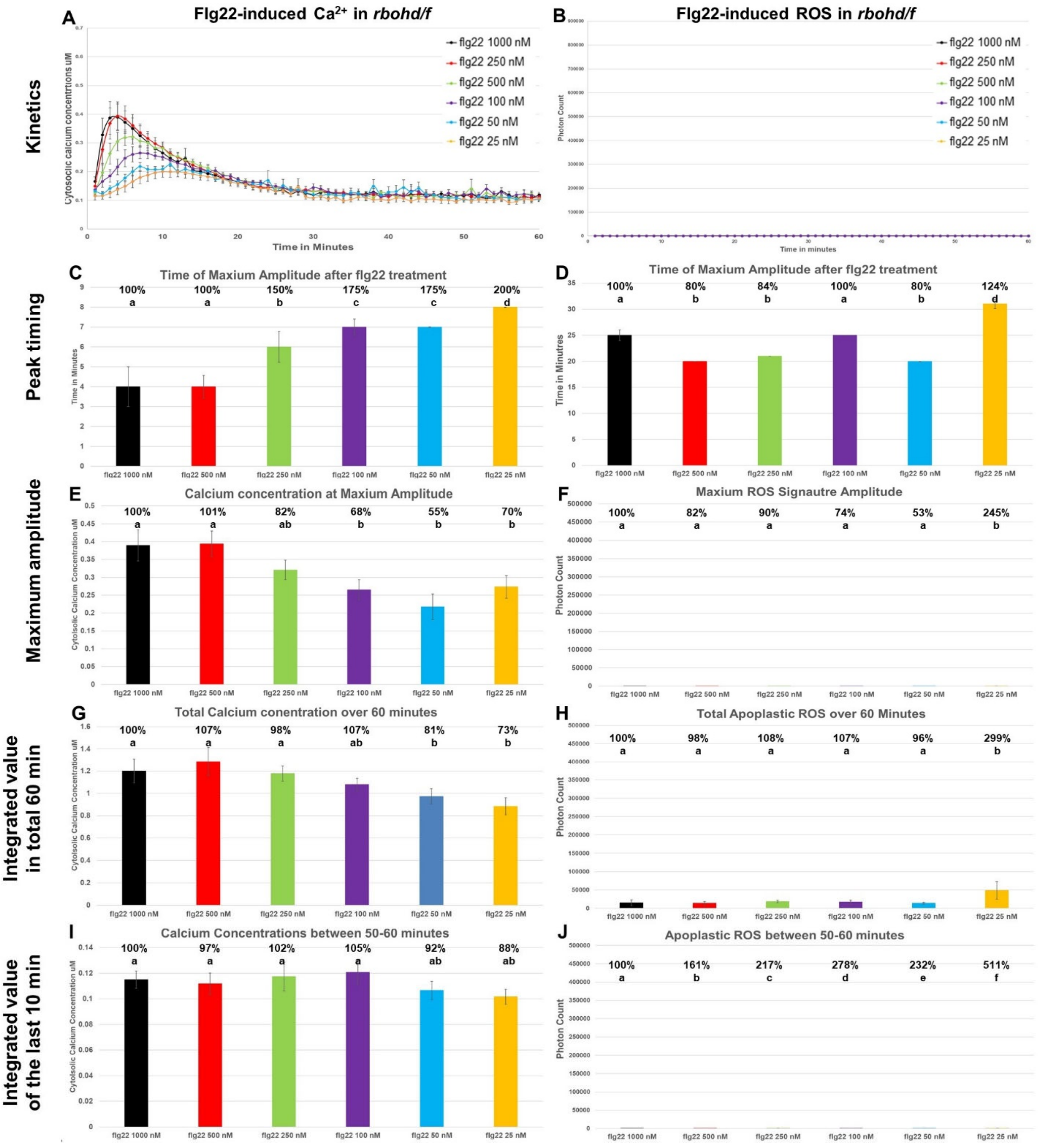

Supplementary Figure S11. Effects of different concentrations of flg22 on cytosolic calcium elevation and apoplastic ROS production in a double knockout mutant, *rbohd/f*. Line graphs show dynamic changes in cytosolic calcium elevation and apoplastic ROS production over 60 min after flg22 addition (A and B). Histograms represent detailed data as the timing of the maximum amplitude (C and D), the maximum amplitude (E and F), the integrated values over 60 min (G and H), and the integrated values between 50–60 min (last 10 min of the kinetics measured) as the recovery of the second messenger (I and J). Leaf discs (n = 24) were treated by flg22 addition at a final concentration of 25, 50, 100, 250, 500, or 1,000 μM. Histogram values above bars represent percentage compared to non-treated plants. Different letters indicate statistically significant differences at $P < 0.05$ with Tukey-Kramer multiple comparison test.
